# Ciprofloxacin loaded PEG coated ZnO nanoparticles with enhanced antibacterial and wound healing effects

**DOI:** 10.1038/s41598-024-55306-z

**Published:** 2024-02-26

**Authors:** Hussan Ibne Shoukani, Sobia Nisa, Yamin Bibi, Muhammad Zia, Anila Sajjad, Afsheen Ishfaq, Hussain Ali

**Affiliations:** 1 Department of Microbiology, The University of Haripur, Haripur, KPK Pakistan; 2 Department of Botany, Rawalpindi Women University, Rawalpindi, Pakistan; 3 Department of Biotechnology, Quaid-E-Azam University Islamabad, Islamabad, Pakistan; 4 Department of Medicine, FRPMC/PAF Hospital Faisal, Karachi, Pakistan; 5 National Institute of Health, Islamabad, Pakistan

**Keywords:** CIP-PEG-ZnO-NPs, Broad-spectrum, Antibacterial, Cytotoxic effects, Nano-drug delivery system, Biological techniques, Biotechnology, Microbiology, Materials science

## Abstract

Antimicrobial resistance is a worldwide health problem that demands alternative antibacterial strategies. Modified nano-composites can be an effective strategy as compared to traditional medicine. The current study was designed to develop a biocompatible nano-drug delivery system with increased efficacy of current therapeutics for biomedical applications. Zinc oxide nanoparticles (ZnO-NPs) were synthesized by chemical and green methods by mediating with *Moringa olifera* root extract. The ZnO–NPs were further modified by drug conjugation and coating with PEG (CIP-PEG-ZnO-NPs) to enhance their therapeutic potential. PEGylated ZnO-ciprofloxacin nano-conjugates were characterized by Fourier Transform Infrared spectroscopy, X-ray diffractometry, and Scanning Electron Microscopy. During antibacterial screenings chemically and green synthesized CIP-PEG-ZnO-NPs revealed significant activity against clinically isolated Gram-positive and Gram-negative bacterial strains. The sustainable and prolonged release of antibiotics was noted from the CIP–PEG conjugated ZnO-NPs. The synthesized nanoparticles were found compatible with RBCs and Baby hamster kidney cell lines (BHK21) during hemolytic and MTT assays respectively. Based on initial findings a broad-spectrum nano-material was developed and tested for biomedical applications that eradicated *Staphylococcus aureus* from the infectious site and showed wound-healing effects during in vivo applications. ZnO-based nano-drug carrier can offer targeted drug delivery, and improved drug stability and efficacy resulting in better drug penetration.

## Introduction

The poor stability, solubility, and side effects due to uncontrolled target mechanism lead to ineffectivness and resistance to the antibacterial therapies, which encouraged scientists to explore developmental stratagems to counter resilient microbes^1^. Innovative drug delivery systems have high demand in the field of microbiology. Nano-medicines have received extensive attention due to profound physicochemical properties, enhanced uptake, biocompatibility, drug-targeting efficiency, and bio-distribution^2^. Many applications of organic nano-particles such as polymeric (polymeric micelles), lipid-based, liposomes, and inorganic nano-particles like silver, and zinc oxide (ZnO) can be helpful in the field of antibacterial nano-drugs delivery systems in pharmaceuticals following suitable modifications^3^. The use of metallic oxides in the clinical setup is associated with challenges including cytotoxicity of nanoparticles as they carry a positive charge which reacts readily with negatively charged proteins of the bacterial cells. Thus, structural instability of the cell membrane occurs, and further production of reactive oxygen species (ROS) causes its damage^4,5^. Antimicrobial resistance is a basic health issue, due to survival in critical conditions bacteria adopt and develop resistance against antibiotics. Many of available antibiotics have become ineffective as a result of these mdifications. Silver nanoparticle-based drug delivery systems had indicated significant antibacterial effects during topical application as compared to individual drugs or NPs^6^. Cephalexin-loaded PHBV nanofiber also showed enhanced antibacterial effects against MRSA isolated from diabetic ulcers^7^. Polyethylene-glycol (PEG) is one of the most extensively used “stealth” polymer as a facilitator and capping agent in pharmaceutics so it can be very useful to develop a nano-drug delivery system. It has been approved by the United States Food and Development Authority and accepted to be safe in pharmaceutics. Capping with PEG creates a layer of hydration due to its hydrophilic nature and makes a good steric barrier. This steric hindrance helps the nano-formulation to resist the aggregation with adjacent NPs, and also with blood plasma and cell components i.e., protein constituents and immunogenic cells^8^. PEG coating on nanoparticles protects them from opsonization, structural aggregation, and phagocytosis by cells of the reticuloendothelial system of the body. The lack of immunogenicity confers PEG-coated nanoparticles with long-term drug holding, and prolonged release time in circulation systems, which can be beneficial for improved absorption with greater permeation and retaining effect^9^. Biological synthesis can also affect the cytotoxic nature of nanomaterials. *Moringa oleifera* is a member of the miracle-folk-medicinal plant that belongs to the family “*Moringaceae*”. It is commonly grown in different parts of the Indian subcontinent and has importance from a socio-economic perspectives^10^. It is frequently used as medicine to resolve skin problems, a natural food dye and also used to treat lung abnormalities like bronchitis, gastric ulcers, and eye infections, and for treating urinary tract infections by traditional methods. It is commonly called a “wonder plant” due to its health-beneficial properties such as anti-asthmatic activities, potential antimicrobial activity, strong wound-healing effect, high anti-inflammatory ability, anti-diabetic action, anticancer and antitumor activity, and cardiac stimulant^11–14^. Due to its special properties like high nutritional value, cholesterol-reducing activities, anti-oxidant capabilities, and significant anti-hypertensive activities^15,16^ the study was designed to use *Moringa oleifera* root extract for green synthesis of ZnO nanoparticles alongwith chemical synthesis . Further the aims of current investigation were to develop ZnO based modified nanoparticles as an effective remedy to treat skin infections.

## Experimental

### Materials

NaOH, Zinc acetate, PEG (6000), Ciprofloxacin, Ethanol, DPPH, DMSO, Glycerin, L-Aspartic acid were purchased from Sigma-Aldrich. *Moringa oleifera* roots powder was purchased from local market at Rawalpindi. Low ionic strength saline (LISS), Human RBCs, Baby Hamster Kidney cell lines (BHK 21 cell line) were provided by Foot and Mouth Diseases Vaccine Research Center Peshawar Pakistan. Nutrient ager and Nutrient broth were purchased from Oxoid.

### Preparation of nanoparticles

#### Chemical synthesis of zinc oxide nanoparticles (Chem-ZnO-NPs)

The chemical synthesis of ZnO-NPs was carried out by the co-precipitation method^17^. Zinc acetate (1 mM) was dissolved into 100 mL distilled water, and 2 M of NaOH-solution (100 mL) was dropwise added into the zinc-acetate solution with continuous stirring (900 rpm) at 60 °C for 3 h. At the end of the reaction, a milky color solution was obtained. The white precipitates were collected by centrifugation at 2000 rpm for 10 min. To remove impurities, precipitates were washed three times with distilled water. The precipitates were dried overnight at 60 °C in a hot air oven and calcinated at 500 °C for 2 h in a muffle furnace.

### Preparation of *Moringa oleifera* roots extract

*Moringa oleifera* roots were commercially purchased. 10 g of root powder was soaked in 90 mL distilled water and stirred continuously for 24 h. The supernatant was separated by centrifugation at 3000 rpm for 15 min at 25 °C. The attained supernatant was separated and stored for further use.

### Green synthesis of Zinc oxide nanoparticles (Green-ZnO-NPs)

ZnO nanoparticles were produced using *Moringa oleifera* root extract as a reducing agent. Briefly, 100 mL of the extract was pre-heated at 60 °C on a magnetic stirrer for 10 min. 5 M solution (100 mL) of zinc acetate dehydrate was added to the above-mentioned extract under continuous stirring (900 rpm) for one hour followed by centrifugation as stated earlier^18^.

### Fabrication and functionalization of ZnO-NPs by PEG capping and CIP loading

PEG (4 mM) was dissolved in 40 mL of distilled water and kept for a day at room temperature. 20 mL of PEG solution was added to flasks separately containing chemical and green synthesized ZnO-NPs (0.1 g/10 mL). The mixture was stirred for 2 days to complete the reaction. The PEG-coated ZnO-NPs were obtained by centrifugation^19,20^. CIP-PEG-ZnO-NPs nano-formulation was prepared by adding 50 mL of 0.010 M solution of CIP to PEG-ZnO-NPs and stirred for 24 h at room temperature. The mixture was centrifuged at 2000 rpm to remove unbound PEG and CIP. Nano-formulations (CIP-PEG-ZnO-NPs) were dried under a vacuum and stored for further applications^21–25^.

### Characterization of ZnO-NPs and CIP-PEG-ZnO-NPs

An X-ray diffractometer (XRD) (D/MAX 2550, Rigaku Ltd., Tokyo, Japan) was used to determine the crystal-phase composition of the synthesized NPs. The X-ray diffraction was monitored in the range of 2θ from 10 to 80 wide-angle XRD (using Cu Kα1 radiation, γ = 1.5406 Å) at 40 kV and 100 mA. The crystallinity of nano-materials was estimated using the Scherrer equation. Fourier Transform Infrared (FTIR) spectrometer (model FTIR-6600 type A Italy) was used to determine the functional groups of all synthesized nano-formulations at 500–4000 cm^-1^. Scanning electron microscopy was performed to investigate the size and shape of the nanoparticle by scanning electron microscopy (MIRA3-TESCAN) Czech Republic. Energy Dispersive Spectroscopy (EDS) was performed to analyze the chemical composition and to evaluate the zinc content in chemical and green synthesized nanoparticles.

### Determination of encapsulation efficiency

The efficiency of drug encapsulation was determined following Liu’s protocol^26^. The nanoparticles were isolated from CIP-PEG-ZnO-NPs by centrifugation at 10,000 rpm for 25 min, and the supernatant was quantified by a spectrophotometer at a wavelength of 295 nm. The encapsulation efficiency (EE) of CIP on CIP-PEG-ZnO nano-particles was determined by the following equation:

$${\text{EE}} = \left[ {{\text{Loaded CIP}} - {\text{PEG}}/{\text{Total amount of nano}} - {\text{formulation }}\left( {{\text{free }} + {\text{ loaded}}} \right)} \right] \times {1}00$$

### Isolation and identification of bacterial strains

Samples of bacterial strains were isolated from clinical samples. Microscopy was performed for bacterial morphology and the standard API20E test panel was used for the biochemical-base identification. Isolated bacterial strains were preserved in glycerol.

### Antibacterial activity

Chemically and green synthesized CIP-PEG-ZnO-NPs and ZnO-NPs alone were used to determine the antibacterial effects by disc diffusion method^27^. To prepare the sensitivity discs, first, the solubility of ZnO-NPs was checked by dissolving them into distilled water and DMSO. ZnO-NPs were soluble in DMSO solution which was coated onto sterilized discs at the concentration of 5 µg/mL. To evaluate the antibacterial activity the bacterial isolates were swabbed on nutrient agar plates separately and the samples’ coated discs were placed on inoculated plates for each strain and incubated at 37 °C for 24 h. As a positive control, Imipenem was used. The zones of inhibition were measured and NPs producing inhibition zones of ≥ 11 mm were considered effective.

### MIC and MBC determination

The minimum inhibitory concentration (MIC) of effective CIP-PEG-ZnO-NPs (exhibited zone of inhibition ≥ 11 mm) was further investigated by the micro broth dilution method. Briefly, the density of bacterial inoculum was maintained at (5 × 10^4^ CFU/mL). Four-fold serial dilutions of test samples (5, 2.5, 1.25, and 0.6 µg/mL) were prepared. Afterward, 195 µL of bacterial culture was added to each well of the 96-well plate and incubated at 37 °C for 24 h. The results were checked by 96-well plate reader and verified by comparing them with positive and negative controls. The last visible clear wells were also subjected to culturing for the determination of minimum bactericidal concentration which showed no growth on the nutrient agar plate^28^.

## Biofilm assays

### Biofilm inhibition assay

200 µL of CIP-PEG-ZnO-NPs (5 mg/mL) were added to the 96-well plate and four-fold serial solutions (5, 2.5, 1.25, and 0.6 µg/mL) were prepared. The positive (broth and bacterial inoculum) and negative control (only nutrient broth) were also added to the plate. After that 10 µL of bacterial inoculum was added into wells excluding negative control wells. The plate was incubated for 6 h for initial cell attachment and biofilm formation. The plate was washed with tap water five times. 100 µL crystal violet dye (1% aqueous solution of dye) was added to each well. After 15 min, plates were washed and allowed to dry for 45 min. In the last, acetic acid (33%) was added to wells, and absorption was taken at 595 nm on a microplate reader. The results were interpreted where color intensity in the wells indicated the presence of biofilm inhibition^29^.

#### Biofilm destruction assay

The potential of CIP-PEG-ZnO-NPs to eradicate pre-formed biofilms was investigated^30,31^. After properly labeling 96 wells plate for each bacterial strain 90 µL nutrient both and bacterial inoculum was added in the first row which was considered as the positive control. The same concentration of bacteria and nutrient broth were also poured in the next rows for each sample with  different dilutions, and plates were incubated. The next day, samples (CIP-PEG-ZnO-NPs) 5, 2.5, 1.25, and 0.6 µg/mL were added in cultured wells and incubated on the plates for 24 h. Later the plates were washed with distilled water and 125 µL crystal violet dye was added into each well and further incubated for 15 min. The microwell plate was washed and covered with tissue paper when it was completely dried (after 2–4) days, 33% acetic acid was added to wells, and a reading was taken at 590 nm using a micro plate reader. Color intensity in the wells indicated the presence or eradication of bacterial biofilm. Each effective nano-formulations were tested against bacterial strains that exhibited sensitivity during initial antibacterial screening.

#### Drug-releasing assay

To determine the drug release profile from CIP-ZnO-NPs and CIP-PEG-ZnO-NPs a Franz diffusion cell was used with a dialysis membrane which has been described by Khan et al.^32^. Percentage release of drug was calculated using spectrophotometer (450 nm) and taking samples at an interval of 2 h till completion of 12 h. Percentage of the drug released was determined by following equation.

$${\text{Percent of drug release }}\left( \% \right) = {\text{Absorbance of sample }}\left( {{\text{nm}}} \right)/{\text{Absorbance of control }}\left( {{\text{nm}}} \right) \times {1}00$$

## Evaluation of antibacterial mechanism of ZnO-NPs and CIP-PEG-ZnO-NPs

### Anti-oxidant assay

Antioxidant activity was determined by DPPH radical scavenging assay^33^. The reaction mixture was comprised of 0.5 mL of NPs (10 µg/mL) and 0.3 mL of DPPH solution which was prepared by dissolving in 3 mL of absolute ethanol. The reaction of DPPH and nanoparticles released oxides resulted in the reduction of DPPH. The absorbance was taken at 517 nm after 30 min of incubation in dark conditions. The negative control was also run by DPPH and ethanol solution mixture. The results are expressed in the percentage of released oxides calculated by the following equation.

$$\% {\text{I}} = {}^{{\text{A}}}{\text{blank }} - {}^{{\text{A}}}{\text{sample}}/{}^{{\text{A}}}{\text{blank}} \times {1}00$$

#### Protein leakage and DNA release assay

The leakage of proteins and DNA from bacterial cells (*Staphylococcus aureus* as Gram-positive and *E. coli* Gram-negative bacteria) was estimated^34^. The Gram-positive and Gram-negative bacteria were mixed with 100 mL LB media and then treated with 10 µg/mL concentration NPs separately. The mixtures were incubated at 37 °C in the orbital shaker at 125 rpm. After 24 h, the culture was centrifuged at 10,000 g for 10 min at 4 °C. The obtained supernatant was used for the estimation of leaked proteins and DNA which were quantified by the nano-drop method.

## Cytotoxicity

### Cytotoxicity of (chemical and green synthesized) ZnO-NPs and CIP-PEG-ZnO-NPs on Baby Hamster Kidney 21 cell lines (BHK21)

To evaluate the cytotoxicity of ZnO-NPs and their CIP-PEG-ZnO-NPs MTT assay was used on Baby Hamster Kidney 21 cell lines (BHK21) provided by Foot and Mouth Disease Research Centre, Veterinary Research Institute, KPK Pakistan^35^. The Baby Hamster Kidney cells were proliferated on Dulbecco’s modified Eagle medium (DMEM) in 96-wells plate and then 1 × 10^5^ cells were distributed into wells before incubation for 24h at 37^*◦*^C. The viable BHK cells (1 × 10^5^) were treated with NPs at 5, 2.5, 1.25, and 0.6 µg/mL. The culture was further incubated at 37 °C for 24 h. Celecoxib was used as the positive control and PBS as the negative control of the study. Following incubation, 100 µL of fresh DMEM was thoroughly mixed with 10 µL of MTT solution prepared in PBS 1X to replace the existing DMEM. The 96-well plates were incubated again for 4 h. Finally, 0.1 mL of DMSO solution was used to dissolve the formazan crystals in the wells, and the OD for MTT formazan, and BHK cells treated with NPs was taken at 570 nm and 620 nm, respectively. The percentage viability was calculated using the given standard equation:

$${\text{Percent cell viability}} = \left( {{\text{Control 57}}0{\text{ nm}} - {62}0{\text{ nm}}} \right)/\left( {{\text{Test 57}}0{\text{ nm}}{-}{62}0{\text{ nm}}} \right) \times {1}00$$

#### Hemolytic assay

RBCs of the ‘O’ blood group were collected after informed consent from subjects. The study was performed following relevant guidelines and procedures (Declaration of Helsinki) approved by the institutional bioethics committee of the University of Haripur. RBCs were washed three times with normal saline by centrifugation at 3500 rpm for 30 s to remove debris from the blood sample and RBCs sediments 20% were diluted with LISS to use in the assay.

##### Preparation of low ionic strength saline (LISS)

Stock solutions were prepared by dissolving 42.9 g Na_2_HPO_4_ and 10.2 g KH_2_PO_4_ separately in 500 mL water. A working solution was prepared by dissolving 1.75 g of NaCl, and 18.0 g of Glycine in water. 8.7 mL Na_2_HPO_4_ and 11.3 mL KH_2_PO_4_ were added to make the volume up to 1 L with distilled water. PH was adjusted to 6.7 with NaOH and 0.5 g of Sodium azide was used as the preservative. The prepared LISS had the following properties: (a) Osmolality: 270–285 mmol, (b) Conductivity: 3.5–3.8 ms/cm at 23 °C, (c) pH 6.6−6.8.

##### Assay procedure

One drop of washed RBCs was taken in a new glass test tube and two drops of LISS, and 100 µL of 1 µg/mL NPs were added. The contents were gently mixed covered with aluminum foil, and placed at 37 °C for overnight incubation. Thereafter, the reaction mixture was centrifuged at 3500 rpm for 30 s, the cherry red color represents a positive reaction and colorless appearance in case of negative results. Two controls were also run, a positive control (D/W + RBCs) which showed a cherry red appearance and a negative control (LISS + RBCs) showed a colorless appearance in the supernatant. The absorbance was taken at 570 nm using a micro-plate reader. The hemolysis percentage (%) of human RBCs was determined by the following equation:

$${\text{Percent }}\left( \% \right){\text{ cell viability}} = \left( {{\text{Absorbance of Sample}}{-}{\text{Negative control}}} \right)/\left( {{\text{Absorbance of Positive Control}} - {\text{Negative control}}} \right) \times {1}00$$

#### Formulation of ZnO nanoparticles based nano-therapeutic

CIP-PEG-ZnO-NPs which exhibited the best antibacterial activity and nontoxic effects during ex-vivo studies, were further used to develop a spray for local application. The spray contained two main types of ingredients one active ingredient ciprofloxacin which was coated on nano-particles. The others were inactive ingredients which included PEG-ZnO-NPs, 5% ethanol as solvent to dissolve the nano-formulate, glycerin as a preservative, adhesive agent, for moisturization, as a facilitator in wound healing, and L-aspartic acid as chemoattractant for bacteria^36,37^. For the preparation of nano-therapeutic, the concentration of 10 mg/L NPs was prepared in 5% ethanol solution with proper mixing by stirring for 2 h with random sonication. Then the mixture was chilled for 30 min in the refrigerator at − 20 °C to make the stronger bonding between NPs, PEG, and CIP. This step is necessary because PEG is unstable at high temperatures and can lose its texture from the NP's surface, chilling can enhance its bonding life span. After that 1 mL of glycerin was added for each 10 mL diluted nano-formulation and further mixed for 6 h. 1 mL of L-Aspartic acid solution was also added into the above mixture and stirred further for 2 h (1 mg/L of L-Aspartic acid solution was prepared in 10 mM concentrated NaOH solution). Prepared antibacterial liquid in the form of spray was stored at 4 °C for further use. HPLC analysis was performed on Shimadzu LC-20AD, to evaluate the Ciprofloxacin and L-Aspartic acid ingredients in prepared nano-therapeutic agents. And in-vivo experiments were followed to evaluate the wound healing effects and infection control capability on infected mice skin.

#### In-vivo studies of nano-therapeutic agents on animal model

The in vivo study was carried out according to the ARRIVE guidelines. All experiments were performed following relevant guidelines and regulations in compliance with the guidelines for the care and use of laboratory animals as approved by the National Institute of Health (NIH), Islamabad with No.F.1–5/ERC/2021. In-vivo experiments were performed on approximately 30 g weighted albino mice model. Their skin hair was removed with hair remover gel, and Xylocaine was applied for numbness, after that all area was cleaned with a spirit swab and a 16 mm lengthy wound was given by surgical blade. Experiments were divided into two groups first was treatment I to check out wound healing effects of topical agent and second was treatment II to check out the eradication of skin infection. The wound healing experiment was divided into three different mice groups., The wound of the mouse was first disinfected and then a chemically synthesized CIP-PEG-ZnO -based topical agent was applied (group I), application of green synthesized CIP-PEG-ZnO-based topical agent in the form of spray (group II) on daily bases (each thrust contain 500 µL of 10 mg concentrated nano-therapeutic agent). The 3rd group was controlled without any application of topical agent. The wound's progress was noted for the next ten days. In Treatment-II experimental grouping was the same but it was processed to control infection. The wound was inoculated with *Staphylococcus aureus* and left till the next day for progression into a skin infection. After confirmation of infection, the topical agent was applied by thrusting, and the wound was covered. For the next ten days same dose was continuously applied daily and the progress of wound recovery was observed by measuring the contraction of wound length due to healing. To monitor the eradication of bacteria from the infection site, the random samples were collected with swab sticks and subjected to microbiology culturing. At the end of the in-vivo study, the skin samples were taken from the treatment site and processed for histological analysis by using hematoxylin and eosin (H&E) staining to evaluate the morphological features of repaired skin of experimental and control groups.

#### Statistical analysis

All the assays were done in triplicate, and the findings are presented as mean with standard deviation. The means were further evaluated using analysis of variance (ANOVA) and least significant difference (LSD) at the probability level *p* < 0.05. All data were analyzed using Origin85, SPSS, and Microsoft Office.

#### Ethical approval and informed consent

All experiments involving animals were performed as per ARRIVE guidelines and approved by the National Institute of Health Pakistan and the Institutional Bioethical Committee of The University of Haripur. Human samples were collected and processed following informed consent as per guidelines provided by the Helsinki Declaration.

## Results and discussions

### Physicochemical characterization

#### FTIR analysis

**Figure 1:**
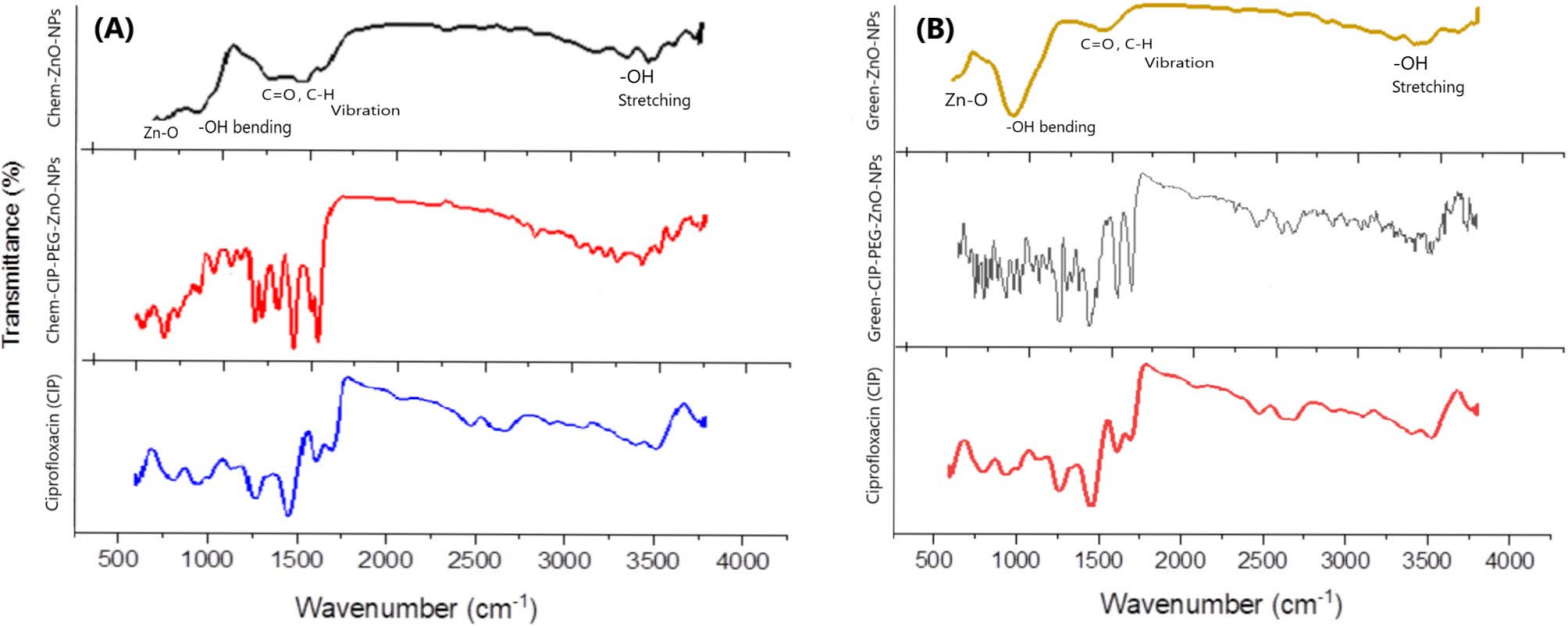
(**A**) FTIR spectra of chemically synthesized ZnO-NPs, pure CIP, and CIP-PEG-ZnO-NPs, (**B**) FTIR spectra of Green synthesized ZnO-NPs, pure CIP, and CIP-PEG-ZnO-NPs.

The chemical conjugation of Ciprofloxacin and PEG with ZnO-NPs was confirmed by FTIR-spectra. The ZnO-NPs spectrum showed characteristic peaks at 3400 cm^-1^ indicating O–H streatching, at 1600 cm^-1^ corresponding C=O (carbonyl) group, and 800 cm^-1^due to the tetrahedral formation of ZnO. The FTIR spectra of ciprofloxacin exhibited characteristic peaks at 3550 cm^-1^ and 1660 cm^-1^. CIP-PEG-ZnO indicates the conjugation of PEG and CIP on ZnO by expressing the same peaks at 900 cm^-1^, 1250 cm^-1^, and 1450 cm^-1^ in both spectra of CIP and CIP-PEG-ZnO (Fig. 1-A).

The FTIR spectrum of green synthesized ZnO-NPs and CIP-PEG-ZnO-NPs is shown in (Fig. 1-B). The characteristic peaks at 3500 cm^−1^, 1660 cm^−1^, 750 cm^-1^attributed to O–H, C = O (carbonyl) groups, and tetrahedral coordination of ZnO respectively as described in previous work. The phenolic compounds in *Moringa oleifera* root extracts can act as reducing agents for the synthesis of ZnO-NPs from zinc acetate^38^. The FTIR spectrum of pure Ciprofloxacin exhibited characteristic peaks at 900 cm^−1^, 1450 cm^−1^, 1600 cm^−1^, and 3560 cm^−1^. The successful conjugation of CIP-PEG-ZnO is represented by characteristic peaks at 1100 cm^−1^and 3200 cm^−1^ for PEG, and at 900 cm^−1^, 1250 cm^−1^, 1440 cm^−1^, 1660 cm^−1^, 2600 cm^−1^, and 3550 cm^−1^ exhibiting CIP-ZNPs patching^39,40^. The results indicated that CIP and PEG are successfully conjugated on green and chemically synthesized ZnO-NPs.

#### XRD analysis

**Figure 2:**
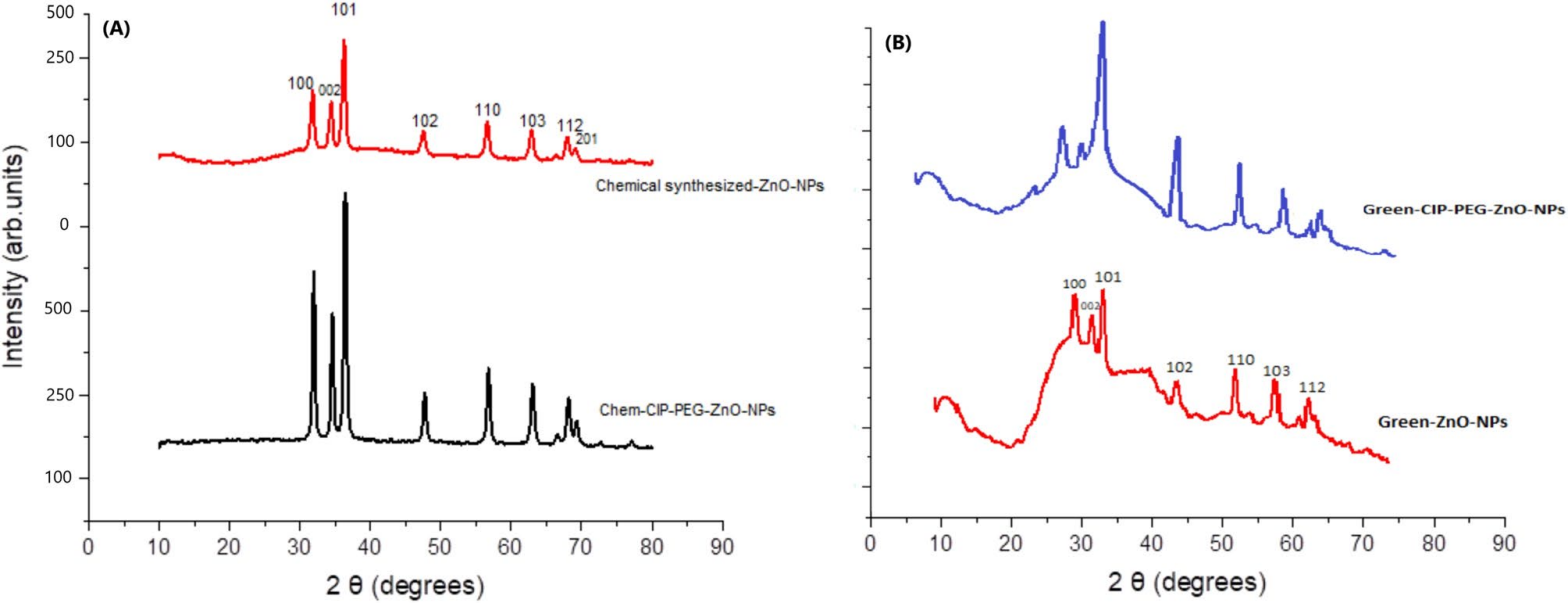
(**A**) XRD pattern of chemically synthesized ZnO-NPs and Chem-CIP-PEG-ZnO-NPs, (**B**) XRD pattern of green synthesized ZnO-NPs and Green-CIP-PEG-ZnO-NPs.

Analyzing the crystalline structure and crystallite size of ZnO directly influences its physical, chemical, and functional properties. XRD analysis was used to provide insight into the crystalline structure and crystallite size of ZnO-NPs and CIP-PEG-ZnO-NPs. The XRD pattern for the chemically synthesized ZnO-NPs showed peaks at 2*θ* = 31.72°, 34.69°, 36.19°, 47.26°, 57.10°, 63.12°, 66.34°, 67.71°, and 69.24°, correspond to the (100), (002), (101), (102), (110), (103), (200), (112), (201), respectively (Fig. 2A).

The XRD pattern of green synthesized ZnO-NPs is shown in (Fig. 2B). The peaks at 2*θ* = 31.93°, 34.82°, 36.41°, 46.95°, 57.61°, 63.42°, and 68.37°, correspond to the (100), (002), (101), (102), (110), (103), (200), (112), respectively that confirms crystal planes with hexagonal structure^40,41^. All peaks were the same in CIP-PEG-ZnO-NPs with high intensity and minor changes which indicated that extra capped material. The average size of the NPs was determined by following the Scherrer equation (D = Kλ/βCosθ). The average calculated size for chemically synthesized ZnO-NPs from the XRD pattern was 52 nm, and Chem-CIP-PEG-NPs was 231 nm. While green synthesized ZnO-NPs, green-CIP-PEG-NPs were 90 nm and 189 nm respectively. The overall size of nanoparticles was increased following the capping of ingredients. X-ray diffraction (XRD), provides insights into the arrangement of atoms, lattice parameters, and orientation, which dictate ZnO's electronic, optical, and mechanical performance. This information is pivotal in modifying ZnO for biomedical applications.

#### Morphological features

##### Scanning electron microscopy (SEM) and energy dispersive spectroscopy (EDS) analysis

**Figure 3:**
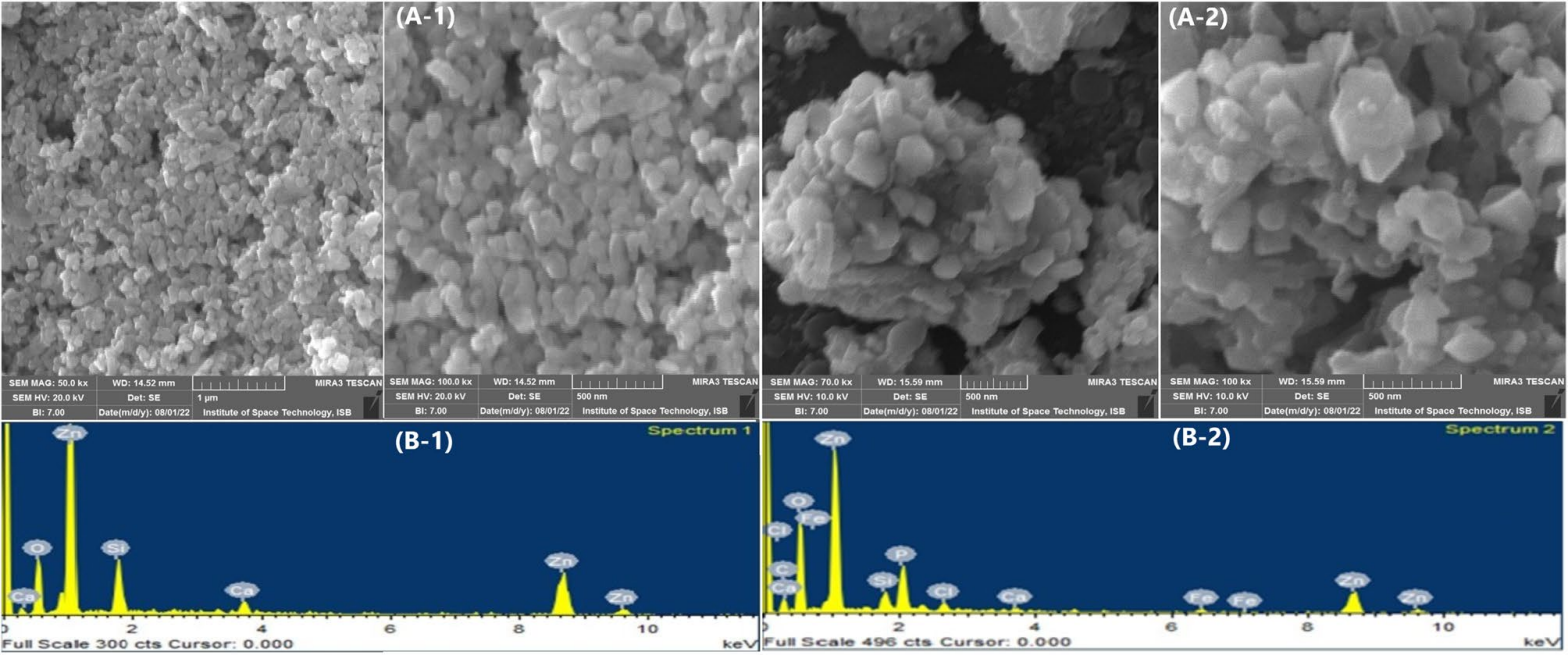
SEM analysis of ZnO-NPs (**A-1**) SEM image of chemically synthesized ZnO-NPs, **(B-1)** EDS of Chem-ZnO-NPs for chemical composition showing the highest peak and percentage of Zinc (**A-2**) SEM image of green synthesized ZnO-NPs, (**B-2**) EDS of green-ZnO-NPs for chemical composition showing the highest peak and percentage of Zinc.

NPs were analyzed by SEM to observe their surface morphological features. The study showed that chemically synthesized ZnO-NPs and green synthesized ZnO-NPs were cylindrical with agglomerates of nano-crystallites. The same surface features of ZnO-NPs were identified by Patra and their co-researchers^42^. During Energy Dispersive Spectroscopy (EDS), NPs showed a high content of Zinc in synthesized NPs as shown in Fig. 3A-1, B-1, A-2, B-2. This analysis aids in determining the uniformity, agglomeration, and dispersion of the nanoparticles, which influence their behavior and are functionally suitable in nano-medicine. This analysis will help optimize synthesis methods by observing the formation process and identifying any irregularities or modifications needed to enhance the desired properties of ZnO-NPs, which contribute to their efficient and tailored utilization in diverse fields.

### Encapsulation efficiency of CIP-PEG-ZnO-NPs

The encapsulation efficiency of chemically synthesized CIP-PEG-ZnO-NPs was found to be 91 ± 1.97% and for green synthesized CIP-PEG-ZnO-NPs it was 93 ± 2.16%**.** Surface charge modifications with PEG enhanced the capping of the drug as earlier reported by Suk et al.^8^. The attachment of ZnO-NPs with Ciprofloxacin can be unstable due to its positive charge, but PEG which is a negatively charged polymer easily capped on the surfaces of NPs with holding drug. PEG capping helps in the tightly holding of CIP with ZnO-NPs and also reduces their cytotoxic effects and makes them biocompatible with long-term drug release^14^.

### Isolation and identification of bacterial strains and, antibacterial effects of ZnO-NPs and CIP-PEG-ZnO-NPs

Bacterial isolates were identified biochemically with a standard API20E test panel and their morphological feature were analyzed through microscopy i.e. Gram-negative identified rods were *Salmonella typhi, Pseudomonas aeruginosa, E.coli, Klebsiella pneumoniae*, Gram-Positive were *Staphylococcus aureus, MRSA* and* Enterococcus faecalis.*

**Figure 4:**
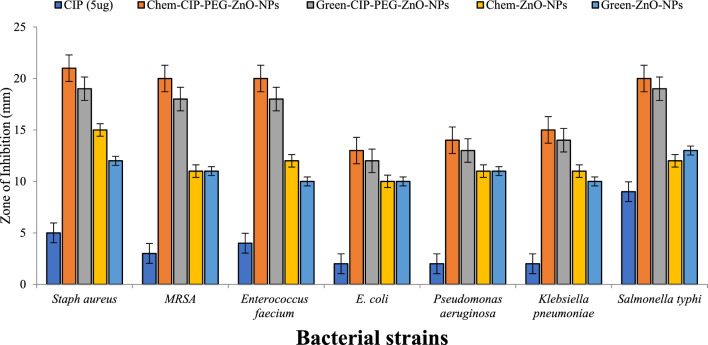
Zone of inhibition of chemical and green synthesized ZnO- NPs/ CIP-PEG-ZnO-NPs against bacterial strains. The values represent the means of replicate experiments, with ± denoting the standard deviation from the mean. Significant differences in values (p < 0.05) are observed.

The antibacterial susceptibility of chemically and green synthesized ZnO-NPs and their nano-formulations was tested by disc diffusion method at a concentration of 5 µg/ml. The results indicated that ZnO-NPs can inhibit the growth of Gram-positive and Gram-negative bacteria with 11 to 14 mm zone of inhibition, the similar results of chemically synthesized ZnNPs are reported by Sharma et al.^27^. In another study, ZnO-NPs were synthesized using *Moringa* leaf extract and their antibacterial capability was lower than our reported *Moringa* root extracts based on synthesized ZnO-NPs^43^. Nano-formulations (Chem-CIP-PEG-ZnO-NPs, Green-CIP-PEG-ZnO-NPs) revealed more effectiveness than individual ZnO-NPs to control the bacterial growth due to synergistic effects of the drug being coated with maximum activity against *Staphylococcus aureus* with zone of inhibition of 21 mm (Fig. 4). Contrary to our findings of the effectiveness of ZnO-NPs at 5 µg/mL, 20 µg/mL concentration of ZnNPs was reported as an effective concentration during antibacterial studies^44,45^. CIP-PEG-ZnO-NPs hold more than 90% encapsulation efficiency which facilitates the long-term binding ability with control CIP release. During the antibacterial activity, CIP-PEG-ZnO-NPs damage the bacterial cell membrane by controlled reduction of their positive charged oxides and alternatively cause the generation of reactive oxygen species (ROS) which attach on cell surfaces as free electrons and in the presence of light create pores^46^. ZnO-NPs are also known to be abrasive due to surface defects which can participate in the physical destruction of bacterial membranes to some extent as well^32,47^.

#### MIC and MBC determination

Effective CIP-PEG-ZnO-NPs that showed > 11 mm antibacterial zones were further subjected to MIC determination by the micro broth dilution protocol. The results displayed the MIC ranging from 1.5–2.5 µg/mLagainst tested bacterial strains. Chemically synthesized-CIP-PEG-ZnO-NPs revealed the MIC value of 1.25 µg/mL against *Salmonella typhi, Staphylococcus aureus, MRSA, Enterococcus faecalis* and 2.5 µg/mL against *E. coli, Pseudomonas aeruginosa* and *Klebsiella pneumoniae.* While Green synthesized CIP-PEG-ZnO-NPs exhibited MIC value of 1.25 µg/mL against *Salmonella typhi, Staphylococcus aureus, MRSA, Enterococcus faecalis* and 2.5 µg/mL against *E. coli, Klebsiella pneumoniae* and *Pseudomonas aeruginosa* (Table 1)*.*

**Table 1:** Determination of MIC (µg/ml) and MBC (µg/ml) against clinical isolates. The values represent the means of replicate experiments, with ± denoting the standard deviation from the mean.

Nano-formulations	*Salmonella typhi*		*E. coli*		*Pseudomonas aeruginosa*		*Staphylococcus aureus*		*MRSA*		*Enterococcus faecalis*		*Klebsiella pneumonia*	
	MIC	MBC	MIC	MBC	MIC	MBC	MIC	MBC	MIC	MBC	MIC	MBC	MIC	MBC
Chem-CIP-PEG-ZnO-NPs	1.25 ± 0.0	2.5 ± 0.0	2.5 ± 0.1	5 ± 0.0	2.5 ± 0.2	5 ± 0.2	1.25 ± 0.0	2.5 ± 0.1	1.25 ± 0.0	2.5 ± 0.0	1.25 ± 0.1	2.5 ± 0.1	2.5 ± 0.1	5 ± 0.1
Green-CIP-PEG-ZnO-NPs	1.25 ± 0.1	2.5 ± 0.0	2.5 ± 0.0	5 ± 0.2	2.5 ± 0.1	5 ± 0.2	1.25 ± 0.0	2.5 ± 0.1	1.25 ± 0.0	2.5 ± 0.0	1.25 ± 0.0	2.5 ± 0.1	2.5 ± 0.2	5 ± 0.0

In the case of MBC Chem-CIP-PEG-ZnO-NPs exhibited MBC of 2.5 µg/mL against *Salmonella typhi, Staphylococcus aureus, MRSA, Enterococcus faecalis* and 5 µg/mL against *E. coli, Pseudomonas aeruginosa* and *Klebsiella pneumoniae* while Green-CIP-PEG-ZnO-NPs exhibited MIC of 2.5 µg/mL against *Salmonella typhi, Staphylococcus aureus, MRSA, Enterococcus faecalis s*and and 5 µg/mL against *E. coli, Klebsiella pneumoniae* and *Pseudomonas aeruginosa* (Table 1)*.* In previous studies CIP-TiO2/Ag/CS nanohybrid showed MIC at 15 µg/mL against *E.coli* concentration while CIP-ZNPs conjugation was effective at 40 µg/mL concentration against *Escherichia coli, Staphylococcus aureus* and *Klebsiella spp*^44,48^.

#### Biofilm assay

**Figure 5:**
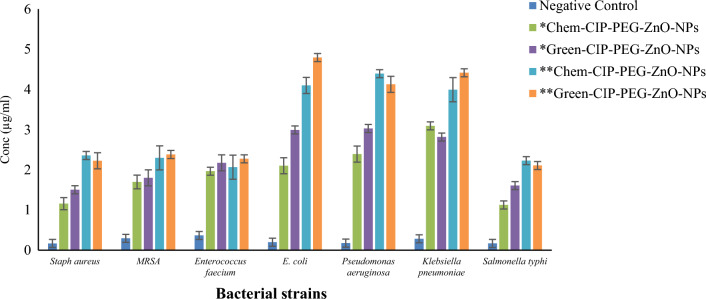
Determination of biofilm inhibition* and biofilm destruction** against clinical isolates. The values represent the means of replicate experiments, with ± denoting the standard deviation from the mean. Significant differences in values (p < 0.05) are observed.

ZnO-CIP-loaded nano-formulates were used to analyze their effects on biofilm formation or inhibition by the micro broth dilution method. Chem-CIP-PEG-ZnO-NPs and Green-CIP-PEG-ZnO-NPs showed anti-biofilm effect against bacterial strains at 1.25 µg/mL concentration for *Salmonella typhi, Staphylococcus aureus, MRSA, Enterococcus faecalis* and while 2.5 µg/mL concentration was effective against *E. coli, Pseudomonas aeruginosa,* and *Klebsiella pneumoniae.* Similarly, biofilm destruction was observed at 2.5 µg/mL concentration against *Salmonella typhi, Staphylococcus aureus, MRSA,* and *Enterococcus faecalis.* In the case of *E. coli, Klebsiella pneumoniae,* and *Pseudomonas aeruginosa* biofilm disruption was observed at 5 µg/mL (Fig. 5). ZnO-NPs of CIP-PEG-ZnO cause their effects by attachment and release of ROS thus damaging the biofilm and bacterial cell membrane which allows the antibiotic-conjugated NPs for deep penetration and disruption of cell division which is not possible with the antibiotic alone^49^. PEG-coated nanoparticles with prolonged drug holding can be beneficial to eradicate the biofilm because of improved absorption with greater permeation and retaining effect. Polyethylene-glycol is a backbone in the fabrication of controlled nano-drug delivery systems even though they require high doses of CIP with prolonged action periods^50^. Anti-biofilm capability of CIP-PEG-ZnO-NPs offers substantial benefits in combating persistent infections. This provides targeted delivery of ciprofloxacin and zinc oxide directly to the biofilm-affected area, which penetrates the protective matrix and enhances the antibiotic's efficacy against biofilm-embedded bacteria. The presence of ZnO nanoparticles can disrupt the biofilm structure due to their antimicrobial properties, potentially preventing biofilm formation or aiding in its breakdown. The combination of ciprofloxacin and ZnO-NPs could synergistically enhance antibacterial activity, which makes it more effective against biofilm-associated infections. This approach holds promise for effectively addressing chronic infections related to biofilm formation, potentially providing a breakthrough in managing conditions linked to biofilm-associated antimicrobial resistance.

### Drug-releasing assay

**Figure 6:**
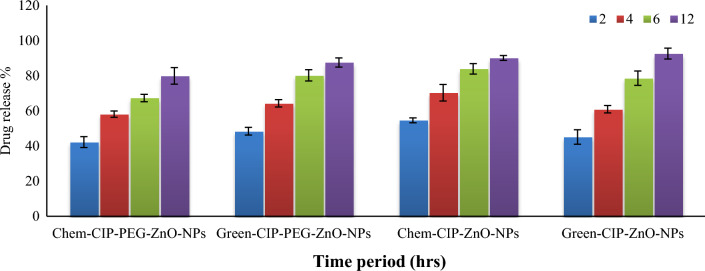
Drug release % from CIP loaded, PEG-coated, and uncoated ZnO-NPs according to the time interval. The values represent the means of replicate experiments, with ± denoting the standard deviation from the mean. Significant differences in values (p < 0.05) are observed.

Percentage of drug release from green-CIP-PEG-ZnO-NPs was calculated as 90.3 ± 1.4% after 12 h, whereas 77.2 ± 1.9% drug release was noted from Chem-CIP-PEG-ZnO-NPs. The drug release from green synthesized-CIP-ZnO-NPs was recorded as 93.4 ± 1.0%, while green-CIP-PGE-ZnO-NPs revealed 85.5 ± 2.0% CIP release after 12 h respectively (Fig. 6). The results indicated CIP release from PEG-uncoated ZnO-NPs was rapid with 90.3 ± 1.4% release of loaded drug in 12 h, whereas PEG-coated ZnO-NPs showed controlled and sustainable release of CIP. In a relevant study, ZnO imprinted dextran hybrid poly (*N*-isopropylacrylamide)/poly ethylene glycol composite hydrogels were formulated to determine in-vitro release of ciprofloxacin, the results indicated 98.2% CIP release in the medium in 6 h^51^. Another study reported enhanced drug release from PEG-coated Ni-FeO-Nanoformulation and showed 1.3% DOX per mint^44^. Evaluation of induced photocatalytic degradation of Ciprofloxacin form ZnO-NPs in an aqueous medium showed 82% release after 160 min^52^.

The controlled release of Ciprofloxacin from polyethylene glycol-coated ZnO-NPs as revealed holds critical significance because PEGylation enhances the stability and biocompatibility of ZnO-NPs, reduces potential toxicity, and can improve their circulation time in the body or drug availability in the target site. This ensures sustained therapeutic levels of CIP and optimizes its efficacy against bacterial infections with minimized adverse effects. This controlled delivery mechanism allows for targeted drug release at specific sites, with enhanced treatment precision and reduced frequency of dosing. Moreover, it helps mitigate the potential development of bacterial resistance by maintaining a constant and effective concentration of CIP.

### Evaluation of antibacterial mechanism of ZnO-NPs and CIP-PEG-ZnO-NPs

#### Antioxidant activity

**Figure 7:**
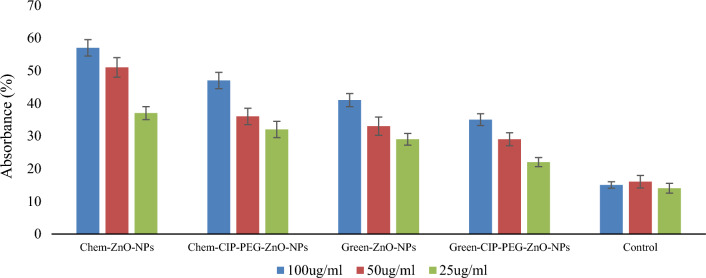
Antioxidant assay of ZnO-NPs and CIP-PEG-ZnO-NPs.

DPPH free radical scavenging assay indicated dose pendent results. The highest free radical scavenging effect was observed for Chem-ZnO-NPs as 57% ± 2.0 at 100 µg/mL and Green-ZnO-NPs showed 41% ± 1.8 inhibition at the same concentration. While Chem-CIP-PEG-ZnO-NPs explored 47% ± 2.0 and Green-CIP-PEG-ZnO-NPs showed 35% ± 1.4 free radical scavenging effect at the highest concentration (Fig. 7). Overall, the DPPH free radical scavenging potential of the NPs decreased in the form of nanoformulations, this was due to the surface capping of particles. The antioxidant activity of synthesized NPs is comparable to other studies performed by Nagajyothi et al.^52^. NPs serve as carriers for therapeutic agents with antioxidant properties, encapsulation of NPs with drugs can enhance their stability, bioavailability, and delivery to tissues or cells. Many drugs and therapeutic agents are sensitive to oxidative degradation, which can reduce their efficacy or even render them harmful. ZnO-NPs, with their antioxidant properties, can help protect these drugs from oxidative damage during storage and transport, and ensure the drugs remain stable and effective until they reach the application. By preventing oxidation, NPs can extend the shelf life of pharmaceutical products by reducing waste and the need for frequent replacements. This is particularly important for biologics and other sensitive drugs. Some drugs may produce harmful reactive oxygen species (ROS) when administered to the body, leading to toxic side effects. ZnO-NPs can act as scavengers of ROS, reducing the potential for drug-induced toxicity and improving the safety profile of these medications. In the form of drug delivery systems ZnO-NPs antioxidant activity can help to maintain the integrity of delivery vehicles, and ensure the drug payload remains protected until it reaches its target site. ZnO-NPs are generally considered biocompatible and have low toxicity when used in controlled concentrations. Drug conjugation with ZnO-NPs can have synergistic antioxidant effects, enhancing the overall therapeutic benefits^53^.

#### Protein leakage and DNA release assay

**Figure 8:**
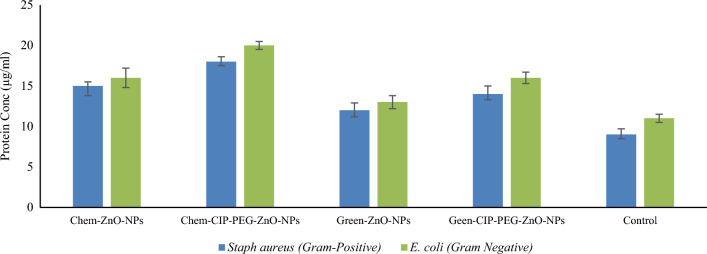
Bacterial protein leakage with the treatment of ZnO-NPs/CIP-PEG-ZnO-NPs

**Figure 9:**
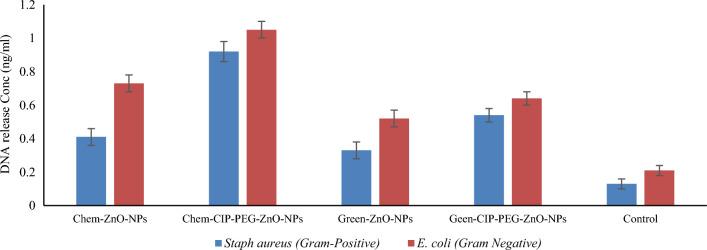
Bacterial DNA release with the treatment of ZnO-NPs /CIP-PEG-ZnO-NPs

CIP-PEG-ZnO-NPs treated bacteria showed a two-fold high released concentration of protein and DNA as compared to individual NPs and control test. It was observed that Chem-CIP-PEG-ZnO-NPs showed 18 ± 0.5 µg/mL and 20 ± 0.6 µg/mL of protein leakage from *Staphylococcus aureus* and *E.coli* respectively, whereas Green-CIP-PEG-ZnO-NPs showed 14 ± 0.3 µg/mL and 16 ± 0.4 µg/mL protein leakage concentration (Fig. 8). DNA release study revealed Chem-CIP-PEG-ZnO-NPs showed 0.92 ± 0.05 ng/mL and 1.05 ± 0.06 ng/mL release concentration from *Staphylococcus aureus* and *E.coli* respectively, whereas Green-CIP-PEG-ZnO-NPs showed 0.54 ± 0.05 ng/mL and 0.64 ± 0.06 ng/mL DNA release respectively (Fig. 9). A relevant study reported 6 µg/mL and 7 µg/mL protein leakage using ZNPs from *E.coli,* and *Staphylococcus aureus* respectively^54^. As compared to the individual ZnO-NPs, CIP-PEG-ZnO-NPs showed good protein and DNA release capability. NPs target the membranal protein structure, where they disturb the physical, functional features of the membrane and create pores, which cause protein and DNA release from the bacterial cell. Membrane damage due to nanoformulations also leads to protein leakage while NPs-CIP interaction with topoisomerase inhibits its activity, and ultimately disintegrates DNA into segments and releases them out from the bacterial cell^55^.

### Cytotoxicity

#### Cytotoxicity of (chemical and green synthesized) ZnO-NPs and nano-formulates on Baby Hamster kidney 21 cell lines (BHK21)

**Figure 10:**
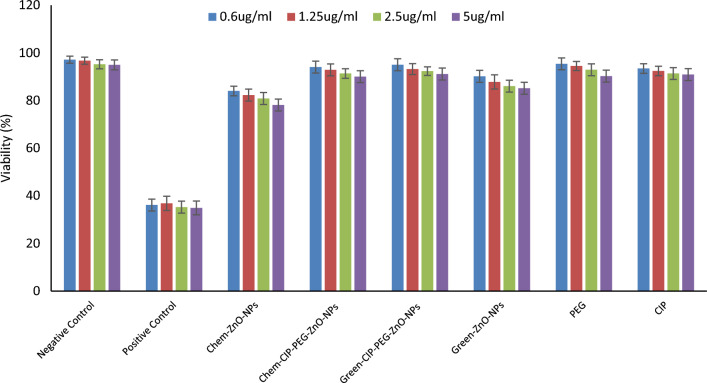
Cytotoxicity of ZnO-NPs and CIP-PEG-ZnO-NPs on Baby Hamster Kidney 21 cell lines (BHK21).

In-vitro cytotoxicity assay was used to observe functionally viable and proliferating cells in a dose-dependent manner. Baby hamster kidney cells treated with Chem-ZnO-NPs exhibited 84 ± 2.2% viability at 0.6 µg/mL and 78 ± 2.0% at 5 µg/mL concentrations while with green Green-ZnO-NPs, BHK cells were found 90 ± 2.5% viable at 0.6 µg/mL and 85 ± 2.2% at 5 µg/mL concentrations. The viability of BHK cells was calculated as 94 ± 2.5% at the minimum concentration (0.6 µg/mL) while 90 ± 2.3% at the maximum concentration (5 µg/mL) of the Chem- CIP-PEG-ZnO-NPs. After treatment of Green-CIP-PEG-ZnO-NPs, the viability of BHK cells was calculated to be 95 ± 2.5% at the minimum concentration (0.6 µg/mL) while 91 ± 2.5% at the maximum concentration (5 µg/mL) (Fig. 10). CIP-PEG-ZnO-NPs cytotoxicity is potentially reduced, ensuring a safer therapeutic profile. Cytotoxic studies on BHK21 cell lines exhibited that CIP-PEG-ZnO-NPs are safer than unloaded ZnO-NPs. The selection of the right nano-material helps to reduce the toxicity. Surface modifications of NPs such as the addition of a biocompatible polymer improves the biocompatibility of NPs and reduces their toxicity towards human cells^56^. CIP-PEG-ZnO-NPs with reduced cytotoxicity hold significant importance in medicine due to their ability to enhance the therapeutic effectiveness of ciprofloxacin while mitigating its potential harm to healthy cells. These types of formulations enable targeted delivery of the antibiotic, which allows precise localization at infection sites while minimizing exposure to non-targeted tissues. This targeted approach not only bolsters the drug's efficacy by improving bioavailability but also holds promise in tackling bacterial infections while potentially curbing the development of antibiotic resistance, making it a valuable strategy in medical treatment.

#### Hemolysis assay

**Figure 11:**
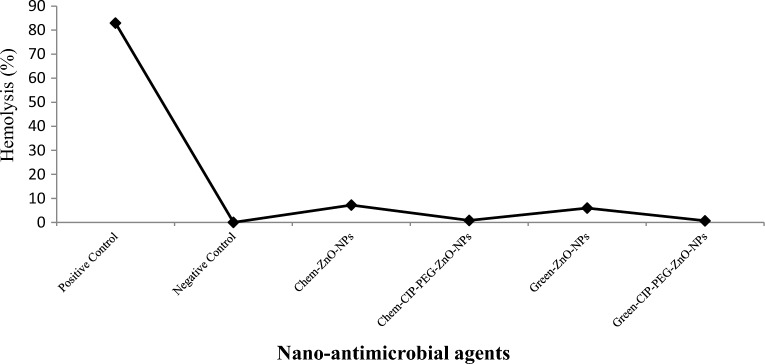
Percentage (%) hemolysis of (ZnO-NPs and CIP-PEG-ZnO-NPs on red blood cells.

During hemolysis studies, Chem-ZnO-NPs showed 7 ± 1.0% hemolysis and Green-ZnO-NPs showed 5 ± 0.8% hemolysis in comparison to positive control which exhibited 82 ± 2.5% of hemolysis (Fig. 11). Whereas treatment of Chem-CIP-ZnO-NPs and Green-CIP-PEG-ZnO-NPs with RBCs did not show any clear hemolysis. Prolonged exposure of RBCs to green and chemically synthesized nanoformulation at 37 °C did not cause any damaging effect as compared to individual ZnO-NPs thus rendering them safe. The use of NPs in medical applications needs to be compatible with the components of blood, especially with RBCs. Incompatibility can lead to adverse reactions and potentially life-threatening complications. Aggregation of NPs can occur when they clump together in the bloodstream, the measures can be taken by PEGylation to prevent NP aggregation ensure smooth circulation, and avoid triggering from immune response^8^.

### High-performance liquid chromatography (HPLC) of topical agent

**Figure 12:**
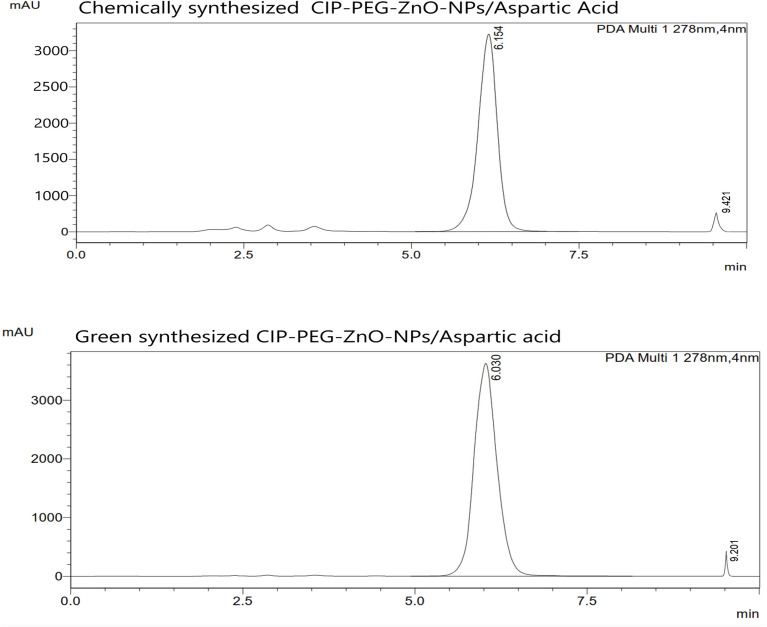
HPLC chromatogram of CIP-PEG-ZnO-NPs based nano-therapeutic agent.

The HPLC results indicated the presence of Ciprofloxacin peak at 6.154 min and for L-Aspartic acid peak at 9.421 retention time in Chem-ZnO-NPs based topical agent corresponding to their target concentration. The Green-ZnO-NPs-based topical agent indicated Ciprofloxacin peak at 6.030 and L-Aspartic acid at 9.201 retention time. Our findings are consistent with previous findings which reported the peaks at the same retention time^57,58^. The chromatogram is shown in (Fig. 12) and the descriptions of the results are in (Table 2).

**Table 2:** Ingredients distribution in HPLC chromatogram of chemically and green synthesized ZnO-NPs based nano-therapeutic agent.

Sample	Substance	Added (mg/L)	Found (mg/L)	Time
Chemical-CIP-PEG-ZnO-NPs/L-Aspartic acid	Aspartic acid	1.0	0.95	9.421
	Ciprofloxacin	10.0	9.5	6.154
Green-CIP-PEG-ZnO-NPs/L-Aspartic acid	Aspartic acid	1.0	0.97	9.201
	Ciprofloxacin	10.0	9.7	6.030

### In-vivo studies of nano-therapeutic agents on animal model

**Figure 13:**
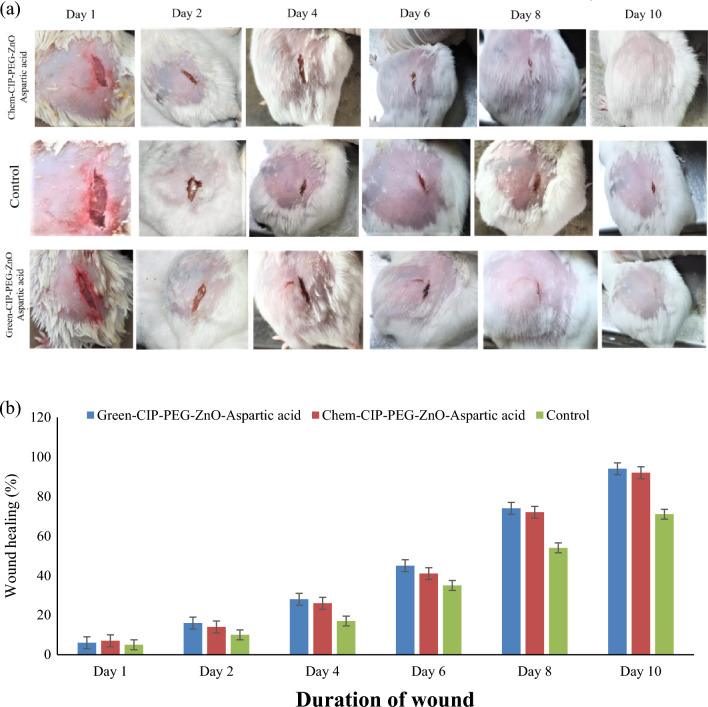
(**a**) Wound healing progress on mice model with the treatment of chemically and green synthesized ZnO-NPs-based nano-drug system (**b**) Wound healing as assessed by reduction of wound size over time.

**Figure 14:**
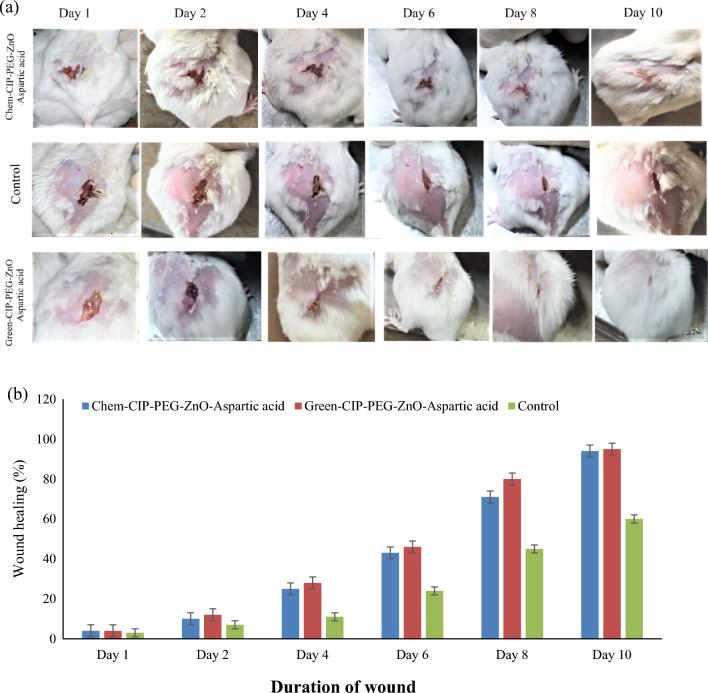
(**a**) *Staphylococcus aureus* infected wound treatment by chemically and green synthesized ZnO-NPs based nano-drug system (**b**) In treatment-II wound was assessed by size and infection clearance over time.

The treatment-I model was processed to determine the wound healing effects of chemically and green-synthesized ZnO-NPs-based nano-therapeutics. The results indicated wound healing progress 20% enhanced as comparison to control group (Fig. 13). The treatment II model was processed to evaluate the eradication of skin infection. It has been observed that chemically and green synthesized ZnO-NPs based nano-therapeutics have ability to kill *Staphylococcus aureus* from infected skin and facilitate the wound healing in comparison to control group. Eradication of bacterial infection was evaluated by culturing of wound swab in microbiology on daily bases which indicated infection control in 2 days as compared to 6 days in case of negative control (Fig. 14; Table 3). There were no physical abnormalities observed in the behavior of mice experimental models and their wound texture was normally healed at the end of treatment. This indicates that synthesized nano-drugs act as effective agent without any side effects during topical application.

**Table 3:** Microbiology culture reports history of treatment II wound swab samples.

Treatment on wound	Day 1	Day 2	Day 4	Day 6	Day 8	Day 10
Chem-CIP-PEG-ZnO-Aspartic acid	+	+	–	–	–	–
Green-CIP-PEG-ZnO-Aspartic acid	+	+	–	–	–	–
Control	+	+	+	+	–	–

Culture with growth (+), Culture with no-growth (–).

#### Histological analysis of treatment-I and treatment-II skin tissues.

**Figure 15:**
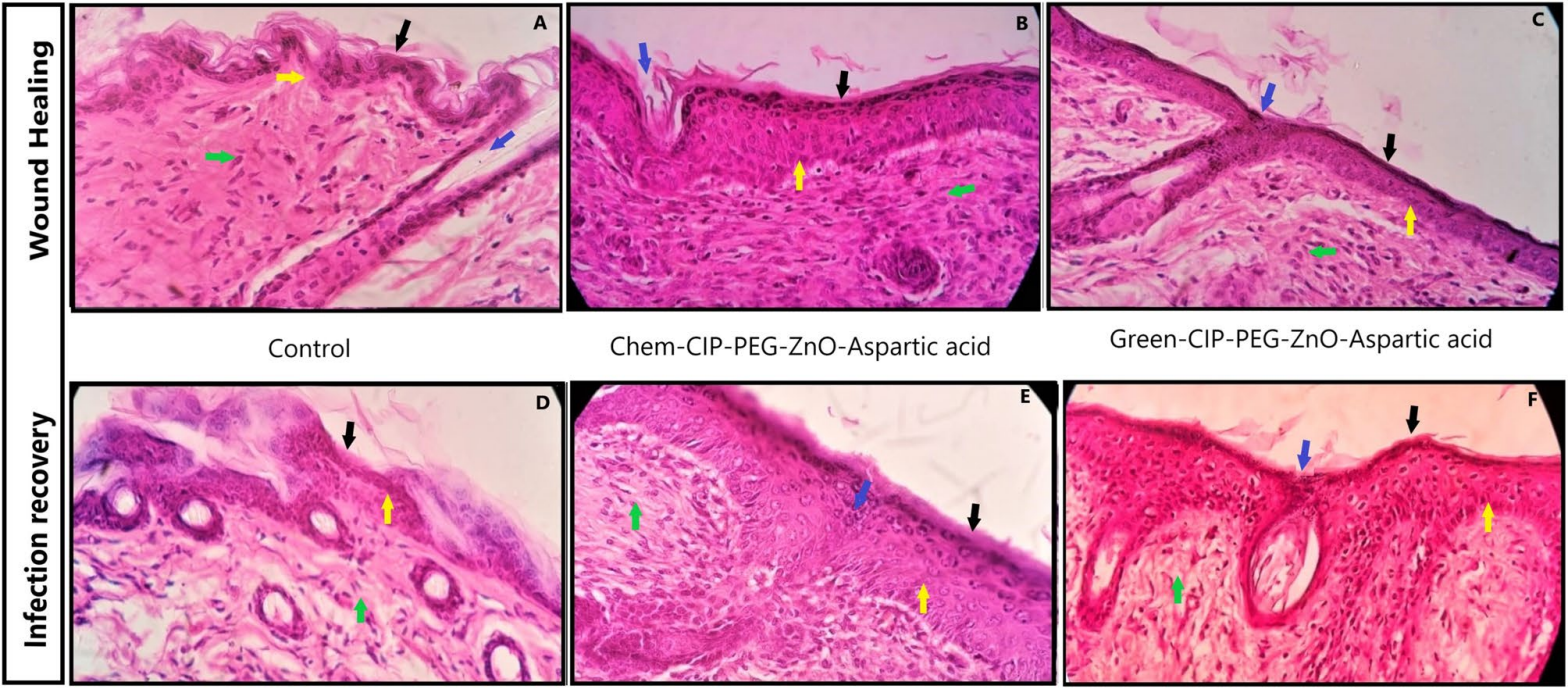
Histological analysis of experimental skin tissues at 40X magnification. (**A**) Wound healing control group without any treatment, (**B**) Wound healing with Chem-CIP-PEG-ZnO-Nanotherapeutic treatment, (**C**) Wound healing Green-CIP-PEG-ZnO-Nanotherapeutic treatment (**D**) Infection recovery control group without any treatment, (**E**) Infection recovery with Chem-CIP-PEG-ZnO-Nanotherapeutic treatment, (**F**) Infection recovery with Green-CIP-PEG-ZnO-Nanotherapeutic treatment. The highlighted figure represents arrows for the Epidermis (Black arrow), Growing hair (Blue), Stratum spinosum of the epidermis (Yellow), Dermis with collagen deposition, and fibroblast regeneration (Green).

Microscopic examination was performed of skin tissues of wound healing and infection recovery groups by using hematoxylin and eosin (H&E) staining on day 10. The tissue of both experimental groups indicated normal regeneration of skin layers and growth of hair follicles after treatment with chemically and green synthesized CIP-PEG-ZnO-NPs based nanotherapeutics. There was no abnormality observed in the cell arrangement or the nuclear structure in either experimental tissue. In the control group of wound healing experimental tissues slow but normal tissue regeneration was observed as compared to the experimental group. The microscopic examination of tissues in the control group of infection recovery revealed irregularity in the cell arrangement of skin layers and neutrophil infiltration was noted which can be due to improper eradication of infectious agents from untreated wounds as shown in (Fig. 15A–F).

Many drugs face challenges related to poor solubility or degradation, limiting their effectiveness^59^. Several NPs showed antibacterial properties but their biomedical application is still pending due to their toxicity to normal cells^60^. In the current study development of biocompatible nano-therapeutic agents with enhanced antibacterial capabilities is a promising candidate to deliver therapeutic agents directly to the site of infection with reducing systemic side effects as also reported by^61^ The outcomes provided sustained release of CIP over an extended period. This controlled release ensures a constant concentration of the antibiotic at the infection site, which can be particularly beneficial for chronic infections of soft tissues^62^. Prolonged release of CIP from nanoparticles can lead to a lower frequency of drug administration in topical treatments. Infected individuals may require fewer doses, which can improve treatment adherence and reduce the burden of multiple administrations. Minimize exposure with non-infected tissues of CIP, non-toxic nanoparticles can reduce the systemic toxicity associated with the antibiotic. Controlled and targeted delivery of CIP to infection sites can help to reduce the development of antibiotic resistance as by maintaining a therapeutic concentration at the infection site, it becomes more challenging for bacteria to develop resistance mechanisms. Effective and compatible drug delivery systems such CIP-PEG-ZnO-NPs can potentially reduce adverse effects and discomfort associated with CIP administration, such as gastrointestinal upset, allowing patients to tolerate the treatment better.

## Conclusion

In this study, ZnO-NPs were synthesized by the chemical and green method using *Moringa olifera* as a reducing agent and formulated into CIP-PEG-ZnO-NPs to develop a biocompatible nano drug delivery system with broad range effects for topical application on skin infections. Both nanoformulations are effective against common pathogenic gram-positive and gram-negative clinically isolated bacteria as compared to drug alone. During in-vitro studies, CIP-PEG-ZnO-NPs were found biologically safe with an acceptable range of toxicity to BHK21 cell line and RBCs as compared to individual NPs. Topical application of modified ZnO nanoparticles can treat skin infections effectively as compared to the control groups without any treatment. Thus the developed modified ZnO nanoparticles offer controlled cytotoxicity with pathogens eradication. The currently designed nanoparticles can help to overcome antibiotics resistance. In future there is a need to test systemic applications and outcomes of these nanocomposites.

## Data Availability

The article contains all the related data and information.

## Abbreviations

CIP: Ciprofloxacin
PEG: Polyethylene glycol
BHK21: Baby Hamster Kidney cell lines
DPPH: 2,2-Diphenyl-1-picrylhydrazyl
MRSA: *Methicillin-resistant Staphylococcus aureus*
CFU: Colony forming unit
MTT: 3-(4,5-Dimethylthiazol-2-yl)-2,5-Diphenyltetrazolium Bromide
NPs: Nanoparticles
ZNPs: Zinc nanoparticles
NM: Nanomaterials
FTIR: Fourier Transform Infrared spectroscopy
SEM: Scanning electron microscopy
XRD: X-ray diffractometry
EDS: Energy dispersive spectroscopy
LISS: Low ionic strength saline
